# A machine learning approach for missing persons cases with high genotyping errors

**DOI:** 10.3389/fgene.2022.971242

**Published:** 2022-10-03

**Authors:** Meng Huang, Muyi Liu, Hongmin Li, Jonathan King, Amy Smuts, Bruce Budowle, Jianye Ge

**Affiliations:** ^1^ Center for Human Identification, University of North Texas Health Science Center, Fort Worth, TX, United States; ^2^ Department of Computer Science, College of Science, California State University, East Bay, Hayward, CA, United States; ^3^ Department of Microbiology, Immunology and Genetics, University of North Texas Health Science Center, Fort Worth, TX, United States

**Keywords:** genetic genealogy, machine learning, genotyping error, feature selection, missing person, hierarchical classification, single nucleotide polymorphisms, kinship estimation

## Abstract

Estimating the relationships between individuals is one of the fundamental challenges in many fields. In particular, relationship.ip estimation could provide valuable information for missing persons cases. The recently developed investigative genetic genealogy approach uses high-density single nucleotide polymorphisms (SNPs) to determine close and more distant relationships, in which hundreds of thousands to tens of millions of SNPs are generated either by microarray genotyping or whole-genome sequencing. The current studies usually assume the SNP profiles were generated with minimum errors. However, in the missing person cases, the DNA samples can be highly degraded, and the SNP profiles generated from these samples usually contain lots of errors. In this study, a machine learning approach was developed for estimating the relationships with high error SNP profiles. In this approach, a hierarchical classification strategy was employed first to classify the relationships by degree and then the relationship types within each degree separately. As for each classification, feature selection was implemented to gain better performance. Both simulated and real data sets with various genotyping error rates were utilized in evaluating this approach, and the accuracies of this approach were higher than individual measures; namely, this approach was more accurate and robust than the individual measures for SNP profiles with genotyping errors. In addition, the highest accuracy could be obtained by providing the same genotyping error rates in train and test sets, and thus estimating genotyping errors of the SNP profiles is critical to obtaining high accuracy of relationship estimation.

## Introduction

DNA-based relatedness estimation is essential for identifying missing persons and human remains. The current standard genotyping technology used in missing person cases (i.e., capillary electrophoresis) measures the lengths of a set of pre-selected short tandem repeat (STR) markers. The major forensic commercial STR kits (e.g., GlobalFiler™ PCR Amplification Kit) usually contain 20 to 25 STR markers, including the core markers defined by the FBI’s National DNA Index System (CODIS) (Hares, 2015). Close relationships (e.g., parent-child and full-sibling) can be determined with high accuracy with this limited number of markers, but not for more distant relationships (i.e., 2^nd^ or higher degrees) (Ge et al., 2011; Ge et al., 2011; Ge and Budowle, 2020). The recently developed investigative genetic genealogy (IGG) approach uses high-density single nucleotide polymorphisms (SNPs) to determine more distant relationships. In this approach, hundreds of thousands to tens of millions of SNPs are generated either by microarray genotyping or whole-genome sequencing (WGS). With the massive number of variants, the distant relationships can be determined with much higher accuracies (Li et al., 2014). Hundreds of missing persons and cold cases have been solved with IGG (Greytak et al., 2019; Tillmar et al., 2021).

The methods to determine relationships with SNPs can be generally classified into three main categories: Hidden Markov Model (HMM) based likelihood ratio methods (LRs) (Boehnke and Cox, 1997; Epstein et al., 2000; Heinrich et al., 2017; Kling, 2019; Galván-Femenía et al., 2021), genome-wide relatedness methods (namely, statistics based on individual SNPs) (Purcell et al., 2007; Manichaikul et al., 2010), and identity-by-descent (IBD) segment detection methods (Gusev et al., 2009; Browning and Browning, 2011; Qiao et al., 2021). The LR methods calculate the likelihoods of the given hypotheses, and the relationship is determined by the maximum likelihood. The LR methods require all loci to be in linkage equilibrium (namely, only a few thousand SNPs may be used) and allele frequencies of each locus are known. The genome-wide relatedness methods summarize the statistic measures from individual markers, and the calculations of these measures are very fast. With a sufficient number of markers, the accuracies are high enough to estimate close relationships (i.e., up to 3rd degree relationships). The IBD segment detection methods use the positions and/or linkage disequilibrium (LD) between markers that the genome-wide relatedness methods ignore and detect the identical haplotype segments shared between profiles, which provide the highest accuracies in estimating relationships, particularly distant relationships. All the methods presume that the SNP calling is accurate with negligible errors (Conomos et al., 2015; Korneliussen and Moltke, 2015; Nøhr et al., 2021; Pew et al., 2015; Shcherbina et al., 2016a; Sherry et al., 2017; Staples et al., 2014; Stevens et al., 2011; Waples et al., 2019). In addition, many of these methods also require allele frequencies or even population admixture ratios in their calculations (Alexander et al., 2009; Thornton et al., 2012; Morrison, 2013; Moltke and Albrechtsen, 2014; Conomos et al., 2015; Conomos et al., 2016).

In missing persons cases, the samples (e.g., bones) can be highly degraded. Thus, the genotyping error rates (GERs) of these samples could be high (e.g., the GER could be 5–10% or higher depending on the quality filtering of the data), and precise allele frequency data are not available. The genome-wide relatedness methods may be more robust to genotyping errors, as the errors at individual markers may not impact the measures of the other markers. However, genotyping errors at one or a few loci can easily interrupt the IBD segments. Thus, the IBD segment detection methods are more sensitive to errors, and their performance may substantially decay as genotyping errors increase. A recent study (Turner et al., 2022) evaluated the impact of GERs on genome-wide relatedness methods and IBD segment methods. The results showed that the overall relationship classification accuracies of different methods were similar if GER is of a low level (GER = 0); however, the accuracies of the IBD segment methods drop quickly when GER is higher than 1% (e.g., the accuracy of hap-IBD (Zhou et al., 2020) approaches to random guessing when GER ≥1%), which means the IBD segments methods are very sensitive to high GERs and require high-quality genotype data. The genome-wide relatedness method (KING) (Manichaikul et al., 2010) had slightly lower accuracies than those of the IBD segment method (IBIS) (Seidman et al., 2020) when GERs were at low-level (i.e., 0 and 0.01). The accuracies of both genome-wide relatedness method and the IBD segment methods, such as IBIS and hap-IBD (Zhou et al., 2020), decreased with higher GERs (i.e., 0.05 and 0.1). However, the accuracies of KING were less impacted by GERs. Thus, more robust methods are needed for missing person samples with high genotyping error. In this study, a supervised machine learning approach was developed for classifying different degrees of relationships and relationship types within the same degrees based on SNP profiles with high genotyping errors. This approach combined 17 genome-wide relatedness measures to train classifiers aiming to reduce the effect of genotyping error and improve the accuracy of relationship estimation.

## Methods

### Simulation data

Family-based genotype data were simulated to train and test the machine learning classifiers. A large pedigree was designed for simulation, which includes ten various relationships from 1st to 3rd degrees and unrelated individuals (Supplementary Figure S1 and Table 1). This designed pedigree was simulated using Ped-sim (Caballero et al., 2019) with the default setting, except that the GERs were specified (i.e., 0, 0.01, 0.03, 0.05, 0.07, and 0.1). GERs larger than 0.1 were not included in the simulation, as the GERs usually could be reduced to below 0.1 with proper quality control and data cleaning, although fewer SNPs would survive (Wall et al., 2014). We first randomly sampled founders and simulated offspring’s genotype data using these founders’ genotype data. In total, 10,000 families were simulated using the pedigree defined in Supplementary Figure S1 (supplementary material). Next, in each simulated family, one pair of each relationship type were sampled (Table 1), and thus the numbers of each relationship in the final dataset were balanced (i.e., each relationship type has the same number of pairs in the training set). The final simulated dataset included 100,000 pairs of individuals with 4 different degrees and 10 different relationship types (including unrelated relationships). Each relationship type has 10,000 instances in the dataset. The simulation adopted 503 unrelated European ancestry (EUR) samples from the 1,000 genomes project sequencing data (30X coverage) (Auton et al., 2015) as founders. For each founder, 582K autosomal biallelic SNPs from Illumina GSA (Global Screening Array) panel were extracted from the 1,000 genomes project and used in the simulation. The GSA panel was selected because most profiles in the genealogy databases (e.g., GEDmatch; https://www.gedmatch.com) were generated by microarray, and GSA is one of the most widely used panels.

**TABLE 1:** The list of 10 relationship types in Supplementary Figure S1.

Relationship type	Relationship degree
Unrelated	N/A
Parent-child	1
Full-sibling	1
Grandparent	2
Half-sibling	2
Uncle-nephew	2
First-cousin	3
Grand-uncle	3
Half-uncle	3
Great-grandparent	3

### Real data

**FIGURE 1:**
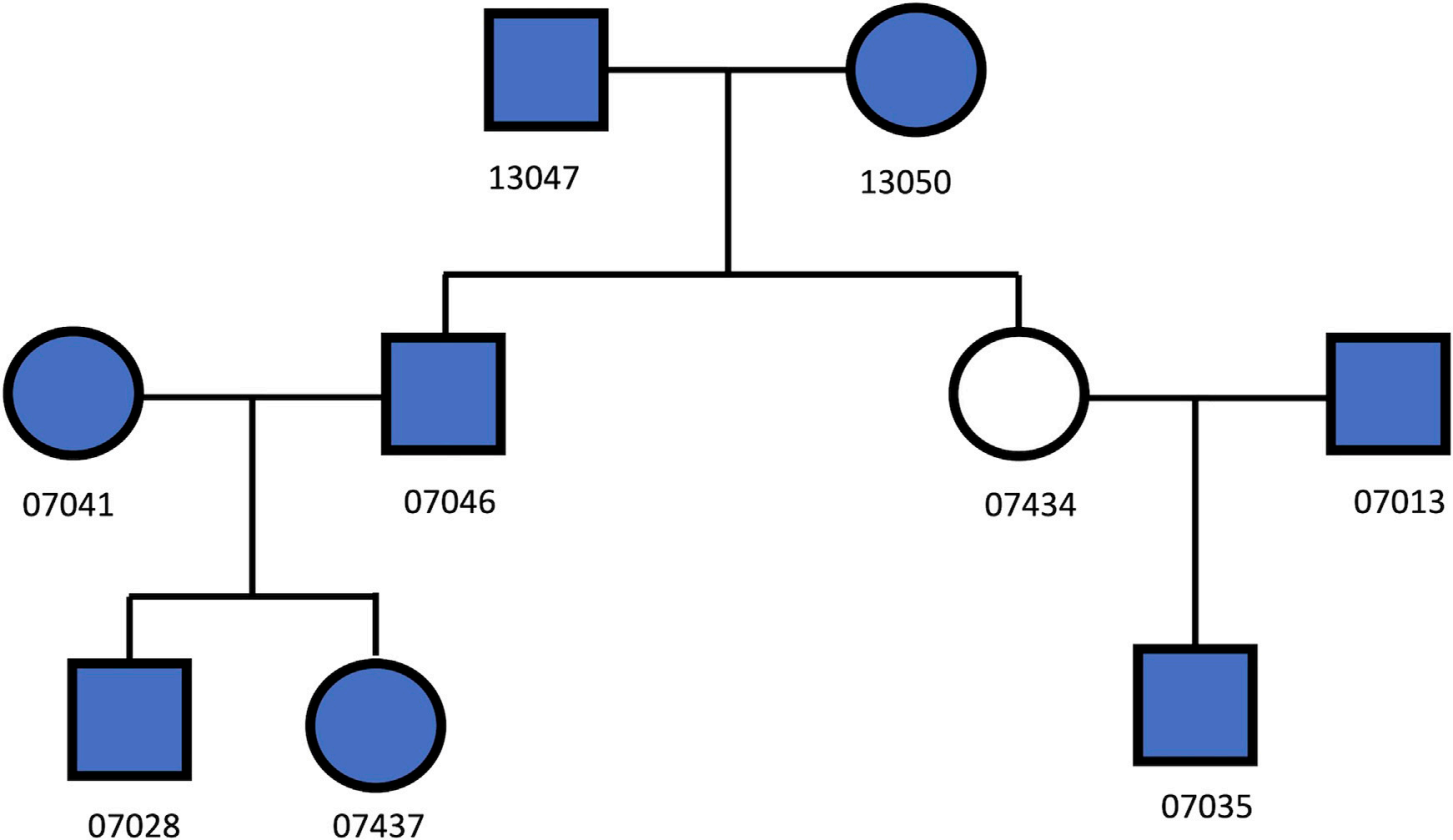
The relationships among the 8 UTAH/CEPH cell line samples (filled in with blue color).

Eight Utah European descendant samples (Dausset et al., 1990) (Figure 1) were selected. Among these samples, there were 28 pairs of relationships, including 11 unrelated, 7 1st degree, 8 2nd degree, and 2 3rd degree relationships. These samples were genotyped using Illumina Infinium Omni5-4 Kit, containing 4.3 million autosomal biallelic SNPs. Each sample was genotyped three times with various input DNA (i.e., 50 ng, 500 pg, and 100 pg). In total, 4,198,873 SNPs were called by Genome Studio, in which 418,513 SNPs were overlapped with the SNPs in the GSA panel. These ∼419 K autosomal biallelic SNPs were extracted and used to test the performance of the trained classifiers.

For the classification with these real data, the simulated datasets with different genotyping errors were used as the training datasets to test the effect of the consistency of the GERs between the reference datasets (assumed to be minimum errors) and the test datasets. For each pair of individuals in the test dataset of classification, a sample containing 50 ng DNA was considered as the reference sample, and another sample containing 500 pg or 100 pg was used as a case sample.

### Feature extraction

For each pair of profiles, either simulated or real data, 17 measures were extracted as features to describe the relationships between individuals (Supplementary Table S1). These measures included KING-homo (K0) (Manichaikul et al., 2010), KING-robust (K1) (Manichaikul et al., 2010), IBS = 0, IBS = 1, IBS = 2, the union of IBS = 0 and IBS = 1 (Stevens et al., 2011), the union of IBS = 1 and IBS = 2, the union of IBS = 0 and IBS = 2, and nine allele combinations of a pair of individuals (j1—j9) (Waples et al., 2019). These features were solely based on the genotypes. Measures with allele frequencies and IBD segments were not included, considering that the allele frequencies of certain populations may not be accurate or even available. In addition, the IBD segment estimation is inaccurate with high error sequence or genotype data generated from degraded samples (e.g., DNA extracted from human bones).

### Classification algorithms selection and hierarchical classification strategy

First, two strong classification algorithms, Random Forest (RF) and Support Vector Machine (SVM), which have different underline learning mechanisms, were compared using 10-fold cross-validation with simulation data to select an algorithm for high classification accuracy and high robustness with noisy data. The higher-performing algorithm would be used for all feature selections and classification. The classification accuracy was determined by 10-fold cross-validations.

**FIGURE 2:**
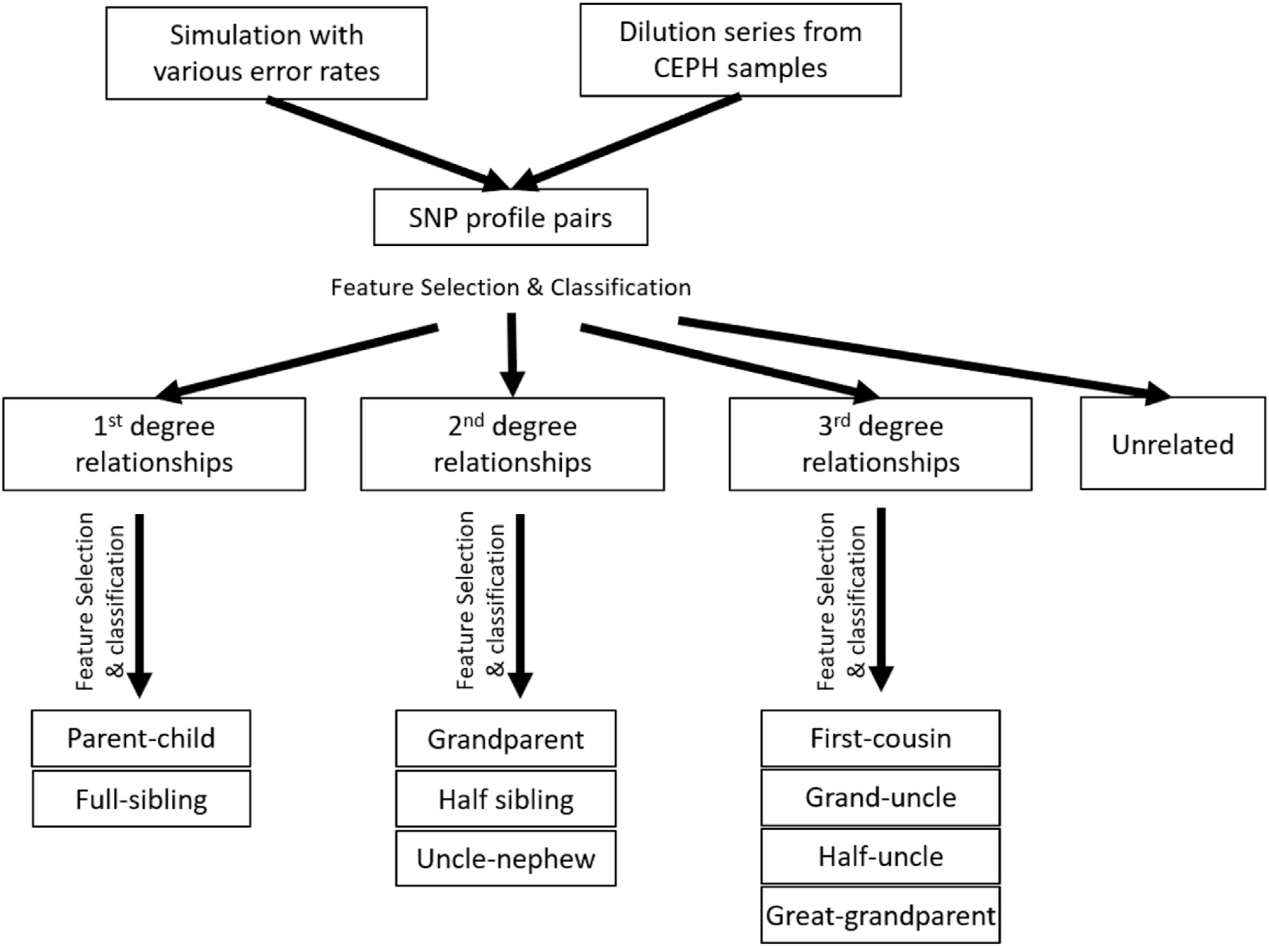
Experimental design and workflow of the whole study. The hierarchical classification was implemented with the simulation data, but not the real data, as the sample size of the real data was too small.

The accuracy of determining the relationship degree is usually much higher than those of determining the relationships within the 2nd or 3rd degrees. The best features to differentiate relationship degrees and relationship types within various degrees may also be different. Thus, a hierarchical classification strategy was implemented to first determine the relationship degree (one classifier) and then determine the relationship type within the same degrees (three classifiers for 1^st^, 2^nd^, and 3^rd^, respectively; Figure 2). In total, there are four classifiers.

For each classifier, forward feature selection was implemented to seek the best performing sets of features for classifications, in which the top-performing (i.e., the highest classification accuracy with 10-fold cross-validation) features among all available features (that have not been selected) were iteratively added to the best performing set using a greedy algorithm. The selected features for a particular classifier may vary with different genotyping errors, as the features may have different degrees of robustness to genotyping errors. The most commonly selected features across all genotyping errors (i.e., the features with the highest robustness and/or the highest classification accuracies) were decided as the final set of selected features. In both real data (i.e., dilution series) and simulation data classifications, the train datasets were the simulated datasets with GER = 0, and the test datasets had various error rates.

## Results

### Supervised classification algorithms comparison

**FIGURE 3:**
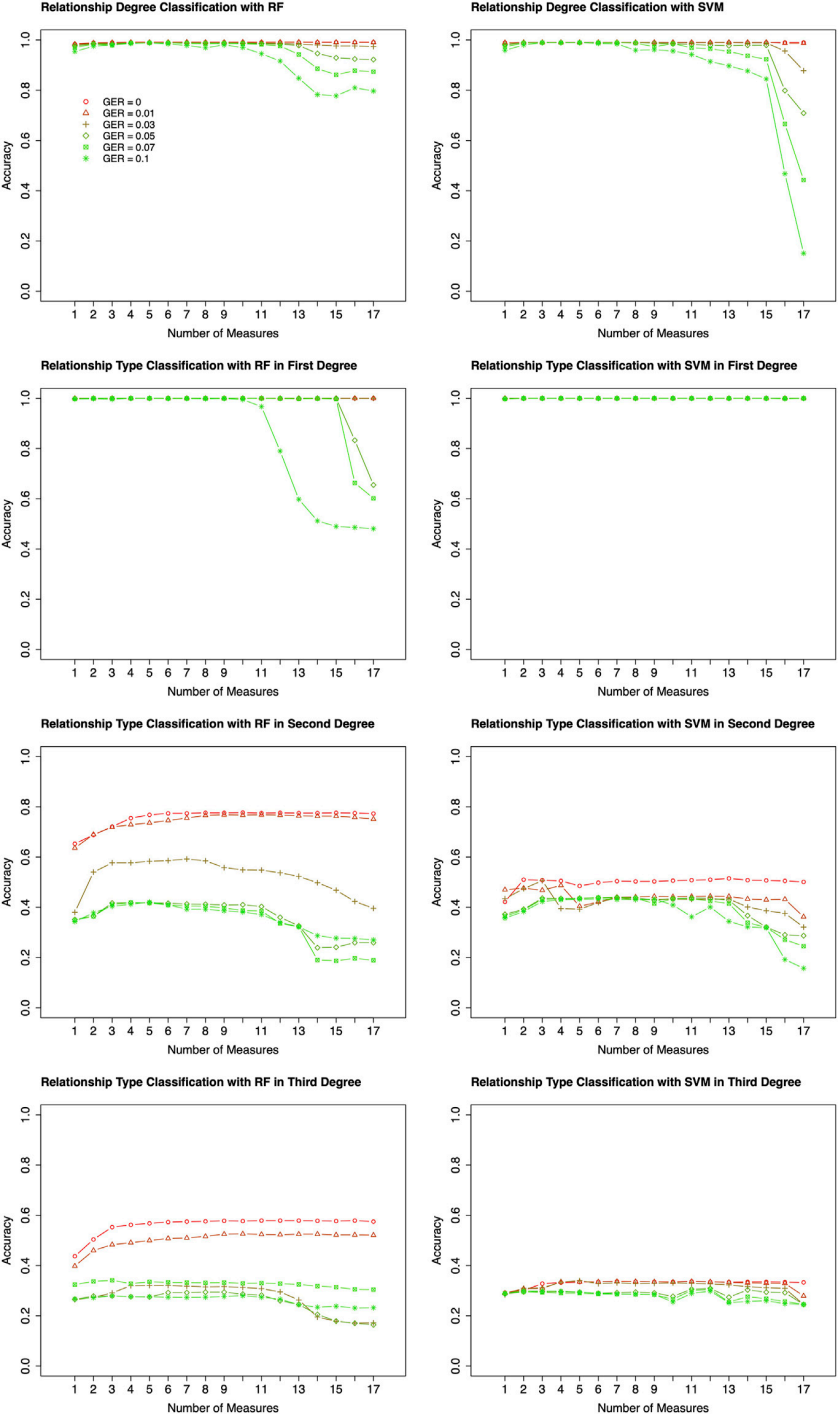
Classification algorithm comparisons between Random Forest (RF) and Support Vector Machine (SVM). Two algorithms (left four plots for RF; right four plots for SVM) were employed to conduct forward feature selection with 10-fold cross-validation for relationship degree, relationship types within the 1st degree, relationship types within the 2nd degree, and relationship types within the 3rd degree. Different genotyping error rates were presented with different colors. The *x*-axis is the number of selected measures (or features) in each step of the forward selection. GER = genotyping error rate of the test dataset.

Based on the results of feature selection and classification (Figure 3), in general, the classification accuracies with RF were higher than those with SVM, except for the classifications of relationship types within 2nd and 3rd degrees with very high GER (i.e., 0.1). For the classification of degrees, both RF and SVM could achieve close to 100% accuracy with the top-performing features, but RF was much more robust. For the classification within the 1st degree relationship, 100% or close to 100% accuracies were obtained with top-performing features across all GERs using SVM (Figure 3). If more than 10 features were selected, the accuracy of RF drops quickly when GER was higher than 0.03, implying that SVM was more robust than RF within the 1st degree. For 2nd and 3rd degree relationships, when the GERs were low (e.g., 0.01), RF could achieve higher accuracies for all three classifications. For example, for relationship type within the 2nd degree, ∼77% accuracy with RF was obtained by the best 8 features, while ∼50% accuracy with SVM was obtained by the best 7 features. In the subsequent analysis, RF with 10-fold cross-validation was employed to conduct forward feature selections and classifications.

### Feature selection

**FIGURE 4:**
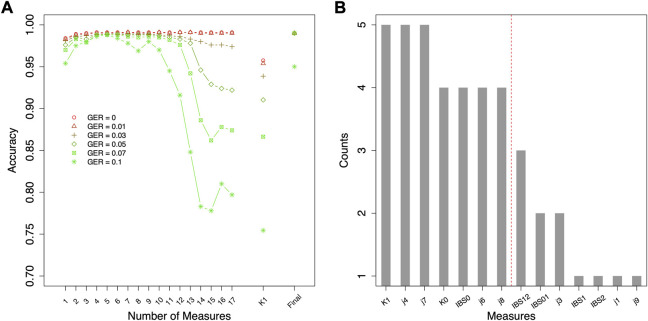
Feature selection for classifying relationship degrees using data simulated with various genotyping errors. **(A)** the accuracies by the forward feature selection with various genotyping errors, in which the ranking of the features for different genotyping error rates (GERs) was different (e.g., the first features were K1 and j7 with GER = 0 and 0.1, respectively), and **(B)** the counts of the commonly selected features across all genotyping errors (e.g., K1 was selected in the feature selections with all six genotyping errors). The features on the left of the red dash line were selected as final features. Final = classification with the selected top-performing features; GER = genotyping error rate of the test dataset.

Different top-performing features might be selected with data simulated using different genotyping errors. For the classification of relationship degrees (Figure 4A), the top 7 features of each given GER would obtain 100% accuracy across all genotyping errors (Figure 4A), and adding additional features would lead to lower accuracies. In total, 42 (= 6 × 7) features were selected across 6 different errors. Figure 4B summarizes the counts of these 42 selected features (14 unique features). K1, j4 and j7 were selected across five genotyping errors, some features were commonly selected (e.g., K0, IBS0, j6, and j8 etc.), and some features were never selected (e.g., j2, j5, and IBS12).

**FIGURE 5:**
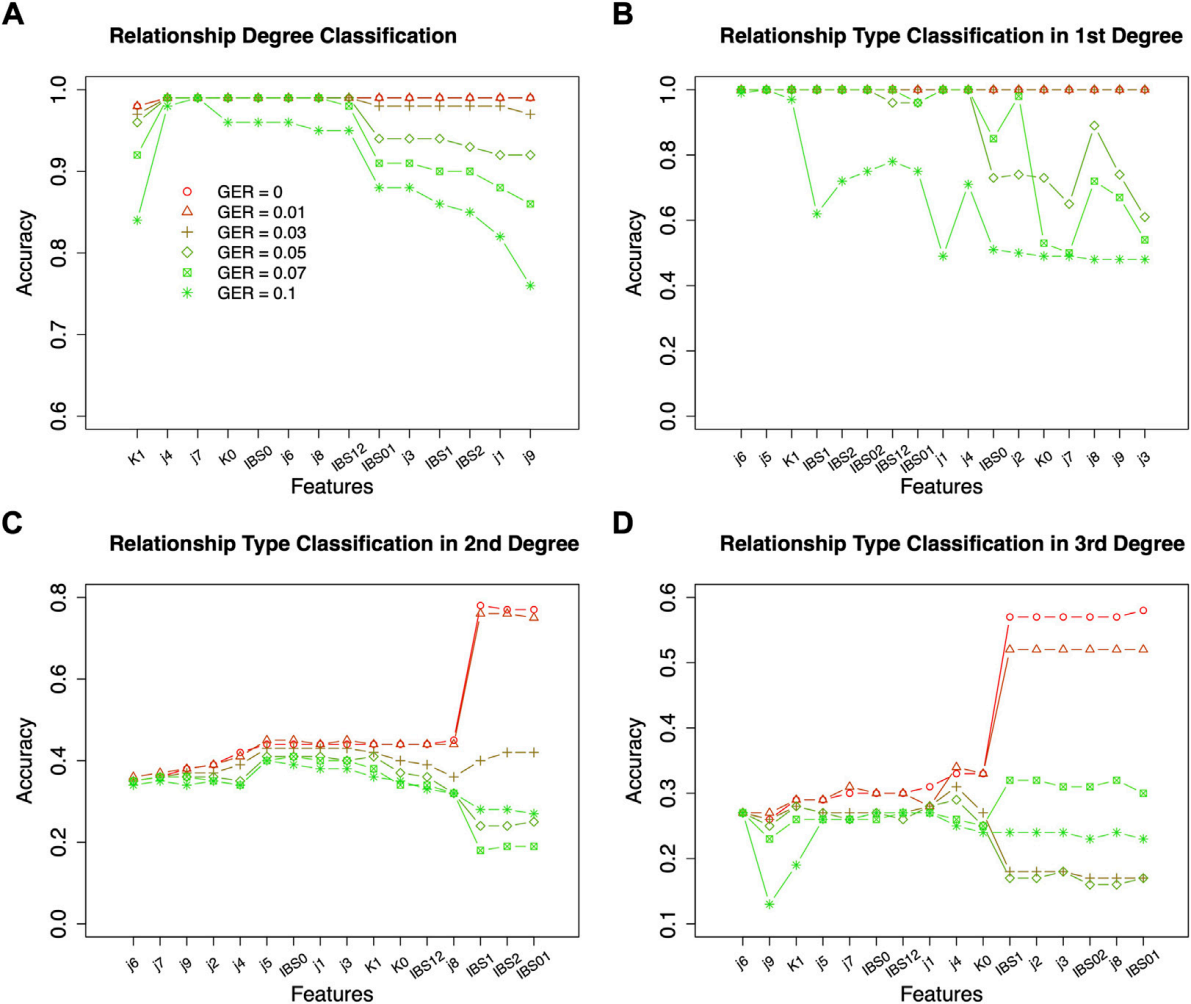
Classification accuracies with the selected features ranked as in Figures 3, 5 for relationship degree and types using data simulated with various genotyping errors. GER = genotyping error rate of the test dataset. **(A)** the accuracies by the forward features selection for the relationship degrees, **(B)** the accuracies by the forward features selection for the 1st degree relationships, **(C)** the accuracies by the forward features selection for the 2nd degree relationships, **(D)** the accuracies by the forward features selection for the 3rd degree relationships.

Since the actual genotyping errors of the forensic samples are unknown or hard to estimate, it might not be appropriate in practice to select different features for different genotyping errors. Thus, feature selection was further conducted by the order of the counts in Figure 4B (i.e., similar fashion as the forward feature selection, but by the ranking of the order of counts) to decide the top-performing set of features that are accurate and improve the robustness across all GERs (Figure 5). For relationship degree classification, the top 7 features (i.e., red dash line in Figure 4B decided by Figure 5A), including K1, j4, j7, K0, IBS0, j6 and j8, were selected as the final feature set because these 7 features had the highest accuracy among all the GERs.

**FIGURE 6:**
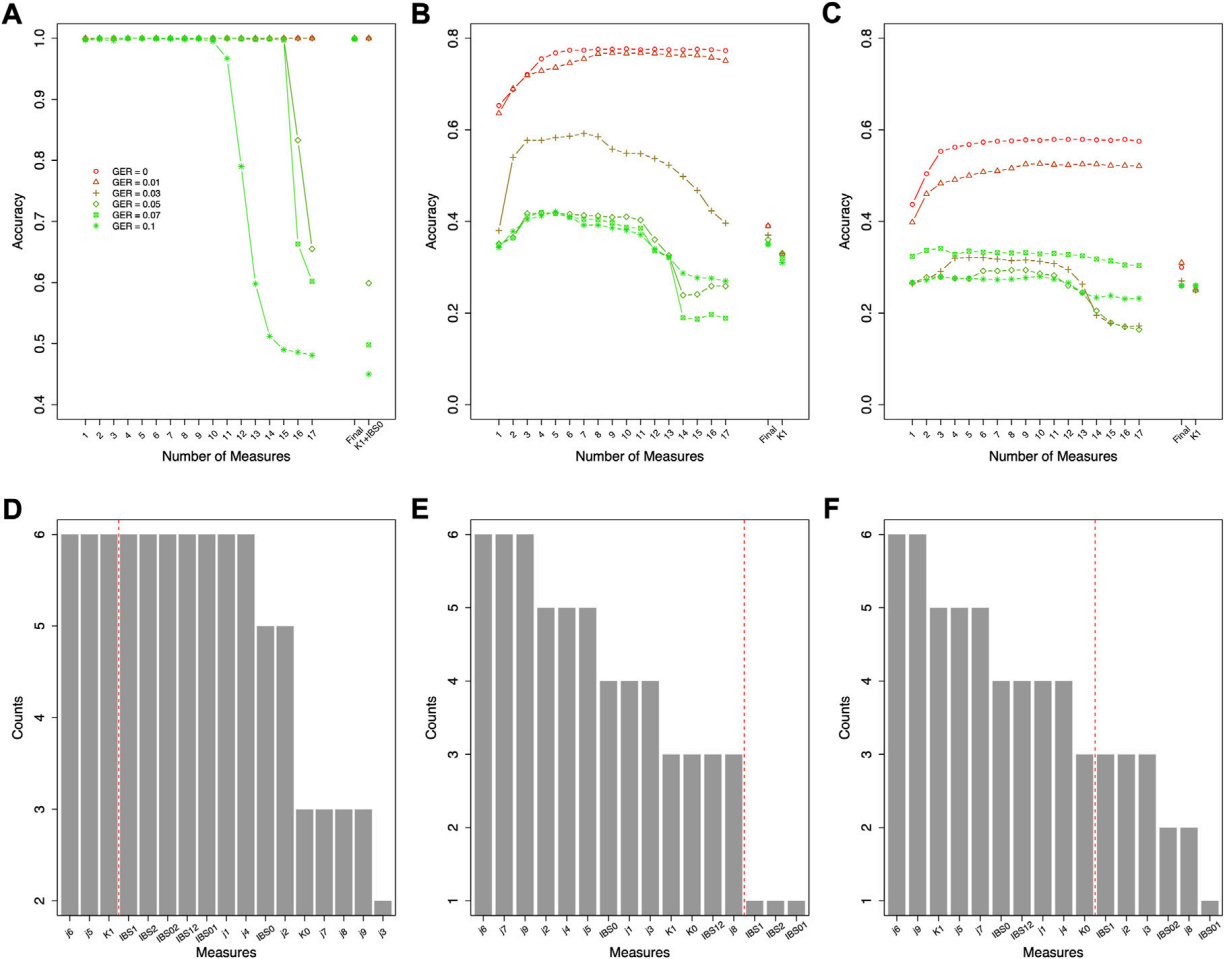
Forward feature selections and classification accuracies for relationship types using data simulated with various genotyping error rates. **(A)** the accuracies for the 1st degree relationships, **(B)** the accuracies for the 2nd degree relationships, **(C)** the accuracies for the 3rd degree relationships, **(D)** the counts of the selected features for the 1st degree relationships across all genotyping errors (e.g., K1 and K0 were selected in the feature selections with all six genotyping errors), **(E)** the counts of the selected features for the 2nd degree relationships across all genotyping errors, and **(F)** the counts of the selected features for the 3rd degree relationships across all genotyping errors. The features on the left of the red dash line were selected as final features. Final = classification with the final features; GER = genotyping error rate of the test dataset.

Similar feature selections were conducted for classifying the relationship types within the 1st degree, the 2nd degree, and the 3rd degree (i.e., the ranking and counts in Figures 6D–F decided by Figures 6A–C). Figures 6D–F summarized the top features across different GERs (the red dash line in Figures 6D–F decided by Figures 5B–D). To balance the classification accuracy and robustness to genotyping errors, we selected the top 3 features for relationship types in the 1st degree, top 13 features for relationship types in the 2nd degree, and top 10 features for relationship types in the 3rd degree (Figures 5, 6).

### Plain classification and hierarchical classification

For relationship degree classification, with the selected 7 top-performing features (i.e., K1, j4, j7, K0, IBS0, j6 and j8), close to 100% classification accuracies were obtained with various GERs (e.g., 99.02% with GER = 0 and 95.24% with GER = 0.1), which was much higher and robust compared with using a single feature K1 (e.g., 95.74% with GER = 0 and 75.45% with GER = 0.1; Figure 4). Apparently, in addition to K1, the other four features in the final feature set substantially improved the accuracy and robustness of the relationship degree classification.

The classification was employed for further classifying the relationship types within each classified relationship degree. The accuracies of classifying the relationship types within the 1st degree were almost 100% with the selected 3 features across all genotyping errors (Figure 6). In contrast, the method suggested in (Manichaikul et al., 2010) to differentiate parent-child and full-sibling (e.g., K1+IBS0) performed very well when genotyping errors were low (e.g., GER ≤0.03), but not for higher GERs (e.g., only 59.9% accuracy with GER = 0.05).

As expected, the classification accuracies within the 2nd degree and 3rd degree were much lower and were substantially affected by the GERs (Figures 5, 6). For the 2nd degree relationship types, the final 13 features together can reach 77.5% accuracy with GER = 0, which was much higher than K1 alone (i.e., 33.3%). The accuracy differences between the final 13 feature set and K1 decreases with increasing GERs (e.g., 32.1% with the final feature set vs. 31.0% with K1 alone, when GER = 0.1). Thus, selecting and combining multiple features could improve the accuracy and robustness, particularly with lower GERs, for classifying relationship types within the 2nd degree.

For the 3rd degree relationships (Figures 5, 6), the accuracies with the final 10 features were higher with different GERs than those with K1 alone. When the GER was low (e.g., GER≤0.05), the final 10 features performed better than K1 alone (e.g., 33.3% with the final feature set vs. 25.2% with K1 alone, when GER = 0). With the GER increasing, the accuracy of the final 10 features could decrease quicker than that with K1 alone (e.g., 24.6% with the final feature set vs. 25.5% with K1 alone, when GER = 0.1). With 4 relationships within the 3rd degree and equal sample size for all relationships, the accuracy with random guessing among the 3^rd^ degree relationships is 0.25. When the GER is high, the selected 10 features perform similar to random guessing.

Overall, the accuracy of classifying relationships within each degree and the robustness to genotyping errors were improved by selecting appropriate features, although the improvement decreases with more distant relationship degrees. As expected, the classifications within the 2nd and the 3rd degree relationships were still challenging. Better features, which contain more than only genotypes, may be needed to improve the accuracy, such as features with allele frequencies in populations.

### Effect of missing data

**FIGURE 7:**
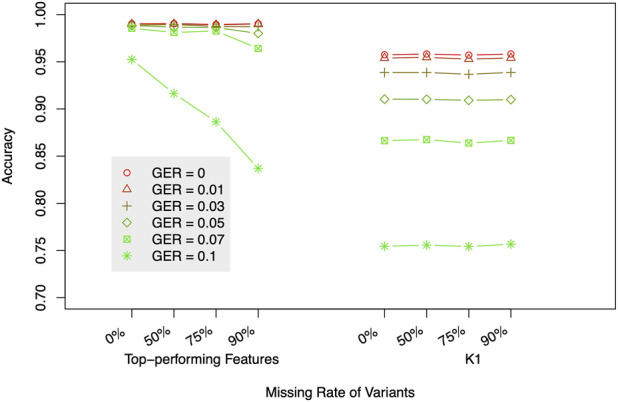
The impact of missing data on relationship degree classification. The label of the *x*-axis represents missing rates with two different sets of features (top-performing features vs. K1). GER = genotyping error rate of the test dataset.

To evaluate the impact of marker density on the classification accuracy, 50, 75, and 90% SNPs were randomly deleted from the 419 K SNPs, and the same analysis as above was conducted on these reduced datasets. Figure 7 compares the accuracies of using 7 top-performing features (Figure 4B) or using K1 only. The accuracies were the average of 10 different runs. Similar to the above analysis, the top-performing features substantially outperformed K1 alone for high genotyping errors. However, the missing data only changed the accuracies when GER is extremely high (e.g., accuracy = 83.7% with ∼42 K SNPs and GER = 0.1), which was consistent with the results in (Manichaikul et al., 2010) and implied that our method is sensitive to SNP density only when GER is very high.

### Effect of various genotyping error rates in the training dataset

In the above studies, the true GER was assumed unknown, and the simulated data with no genotyping error was used as the training dataset. However, if the GERs are known or may be precisely estimated, more appropriate training datasets, which have the same or similar GERs as the test sets, may be used to train the classifiers, and higher accuracies may be obtained.

**FIGURE 8:**
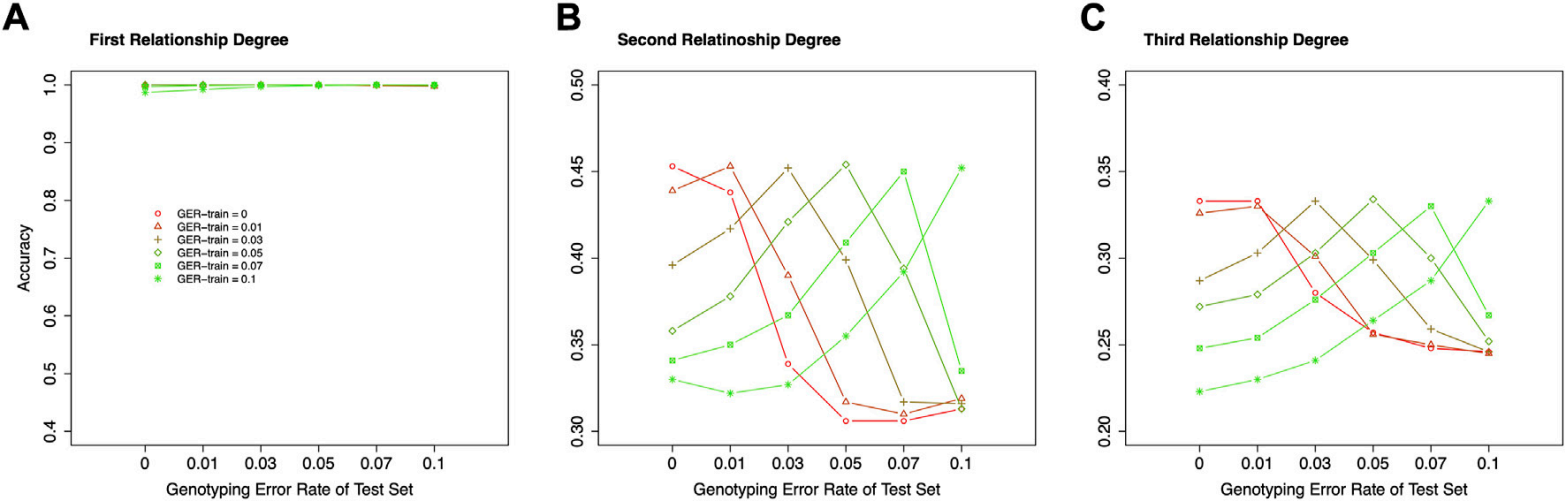
The effect of genotyping error rate of the training dataset in relationship type classification. **(A)** the accuracies with the final selected features for the 1st degree relationships in the training and test datasets with different genotyping error rates, **(B)** the accuracies with the final selected features for the 2nd degree relationships in the training and test datasets with different genotyping error rates, **(C)** the accuracies with the final selected features for the 3rd degree relationships in the training and test datasets with different genotyping error rates. The labels of the *x*-axis **(A–C)** represent the different genotyping error rates of the test dataset. The labels of the *y*-axis **(A–C)** represent classification accuracy. GER-train = genotyping error rate of the training dataset.

Figure 8 shows the accuracies of classifying relationships within the same degrees with various genotyping errors in the training dataset and test set. For the 1st degree relationships, all accuracies were close to 100%, and no impact could be observed. For the 2nd and 3rd degree relationships, the highest classification accuracy always is presented when the train datasets and test datasets have the same GER. Therefore, correctly estimating the GER of a sample could substantially increase the relationship estimation accuracy.

### Relationship degree classification with real data

**FIGURE 9:**
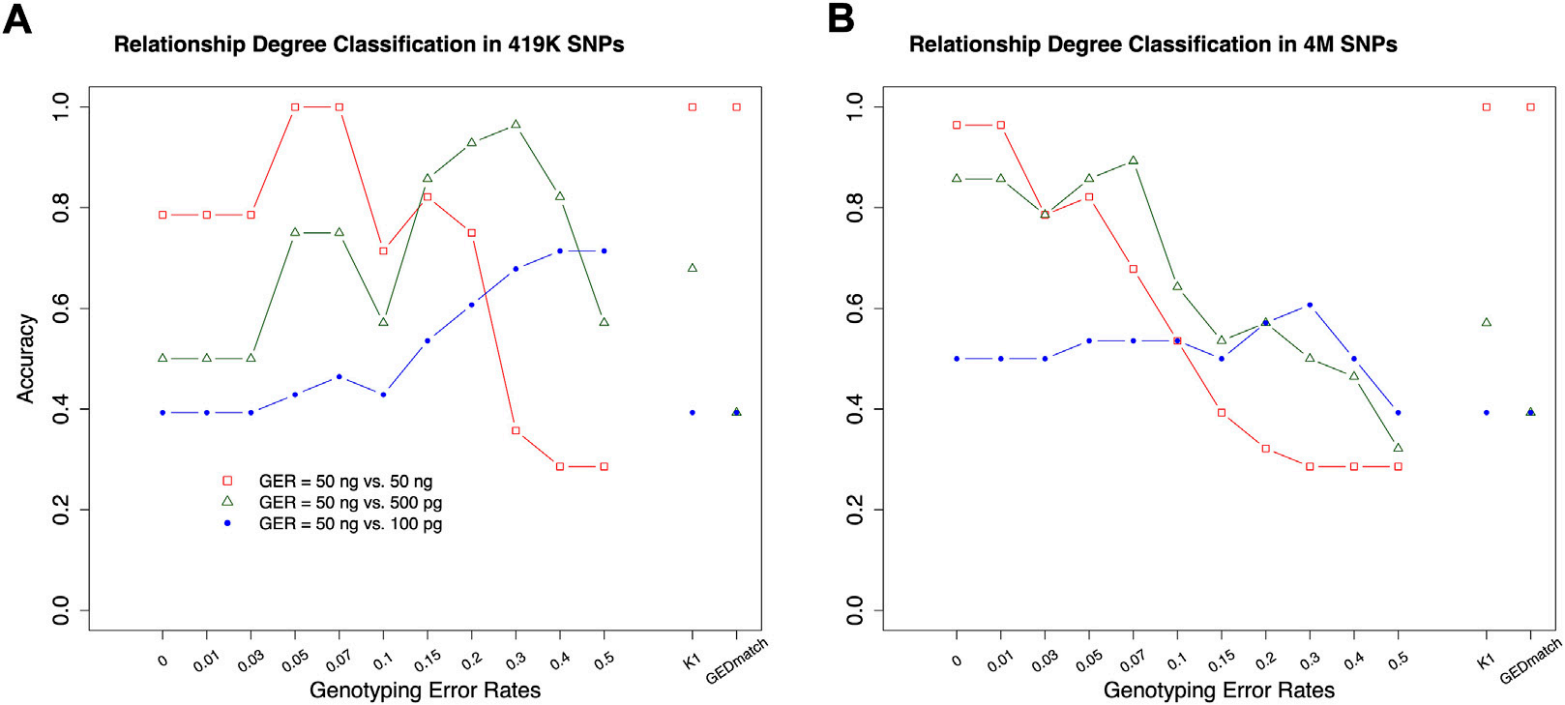
The accuracies of classifying relationship degrees with the UTAH family in Figure 1. The accuracies were estimated with the final selected features for the relationship degrees in the simulated training and real test datasets (UTAH family) with different genotyping error rates. **(A)** test dataset with 419K SNPs, **(B)** test dataset with 4M SNPs. The labels of the *x*-axis **(A,B)** represent the different genotyping error rates of the training dataset. The labels of the *y*-axis **(A,B)** represent classification accuracy. In the legend, GER denotes genotyping error rate of the test dataset. The GERs of the test datasets were represented using different colors and icons. GEDmatch denotes the accuracies obtained from GEDmatch website. K1 denotes the accuracies obtained from KING-robust.

The classifiers, trained by the simulation data with various GERs, were used to classify the pairs in the real samples into degrees, with 50 ng, 500 pg or 100 pg DNA, and ∼4M or 419 K SNPs (Figure 1). K1 with the cutoff thresholds defined in (Manichaikul et al., 2010) was used as the baseline to evaluate performance improvement. In general, the highest accuracies were achieved with close to the true GERs of the test sets (Figure 9). With 50 vs. 50 ng (i.e., both reference and test samples were genotyped with 50 ng DNA), the highest accuracy was 100% (= 28/28) with test sets’ GERs ranging from 0.05 to 0.07 with 419 K SNPs, or accuracy was 96.4% (= 27/28) with GERs from 0 to 0.01 with 4M SNPs. K1 alone achieved 100% accuracy with both 419 K SNPs and 4M SNPs (Figure 9).

With 50 ng vs. 500pg, which may reflect more realistic scenarios in many missing persons cases, the highest accuracies were 92.9% (GER = 0.3) and 89.3% (GER = 0.07) with 419 K or 4M SNPs, respectively. In contrast, the K1 alone only achieved 67.9 and 57.1% with 419 K or 4M SNPs, respectively. Our approach outperformed K1 for high GER profiles generated from low-quality samples. Similar patterns were observed with 50 ng vs. 100pg, in which the highest accuracies were achieved with GER of 0.5 for 419 K SNPs (i.e., 71.4%) and with GER of 0.3 for 4M SNPs (i.e., 60.7%), respectively. The accuracy with K1 alone was only 39.3%. Therefore, higher kinship estimation accuracies could be achieved with our machine learning approach.

In addition, the GERs of these diluted samples might be roughly estimated, as the highest accuracies are likely obtained when the train and test sets share the same genotyping errors (Figure 8). The samples with 50 ng might have a GER lower than 0.03, the GERs of the samples with 500 pg might range from 0.05 to 0.07 if the test with 4M SNPs was considered to be more reliable, and the GERs of the samples with 100 pg might range from 0.2 to 0.3.

To further evaluate the performance of the IBD segment method in real data, we uploaded these 28 SNPs profiles (with ∼4 million SNPs) to GEDmatch. GEDmatch only takes SNPs in the GSA panel in calculation, thus only ∼419 K SNPs were used in calculation. As expected, the accuracy of GEDmatch for profiles generated with 50 ng DNA was 100% (Figure 9 & Supplementary Table S2), as these profiles had minimum genotyping errors. However, the accuracies of GEDmatch were much lower for profiles with 500 and 500 pg DNA, compared with the machine learning approach. For example, the total IBD segments between samples 13,047 and 7,046 dramatically decreased with the reduction of DNA concentration (i.e., 3,571.1 cM with 50ng, but 0 cM with 500 and 100 pg). With 500pg, 15 related pairs (out of 17) were determined as unrelated (i.e., 0 cM), one 1st degree pair was determined as 3rd degree, and 1st degree pair was determined as 8th degree. With 100 pg, 16 related pairs were determined as unrelated, and one 1st degree pair was determined as 3rd degree. If unrelated pairs were excluded in comparisons, the accuracies of GEDmatch for profiles with 500 and 100 pg were 0%. Thus, for profiles with high genotyping errors, GEDmatch may not be a good tool to search the true relatives.

## Discussion

This study developed a novel machine learning approach for estimating the relationship types with high error SNP profiles. In this approach, a hierarchical classification strategy was employed first to classify the relationships by degree and then the relationships within each degree separately. For each classification, feature selection was implemented to gain better performance. Both simulated and real data sets were utilized in evaluating this approach, and the accuracies of this approach were higher than K1 alone (the most commonly used measure) and also other individual measures; namely, this approach was more robust than individual measures for SNP profiles with genotyping errors. In addition, the highest accuracy could be obtained by providing the same GERs in the train and test sets, and thus estimating genotyping errors of the SNP profiles is critical to obtaining high accuracy of relationship estimation.

The accuracy for estimating the degrees of the relationships was close to 100% using simulation data, which showed that the feature selection could be helpful in improving the robustness of classification. K1 was sensitive to genotyping errors, particularly when the GER was higher than 0.07 (Figure 4). Adding more features could substantially improve the accuracy (i.e., close to 100%) and the robustness to errors, which implies that most of the errors in kinship estimation due to genotyping errors could be corrected by adding other features.

The accuracy of estimating the degrees of the relationships obtained from the real data was much lower than the simulated data, which indicated that the simulation model used in Ped-sim might not precisely reflect the genotyping errors in the real data, and thus may result in overfitting in the training dataset. Better genotyping error models (de Vries et al., 2022; Nagraj et al., 2022) need to be developed to simulate SNP profiles that better approximate real SNP profiles generated from low-quality samples. However, in the simulations, the most important issue may be “what error rate should we assign to each type of error defined in the model?” As far as we know, limited studies have been done to generate empirical data for estimating the error rates (or the range of the error rates) for different types of genotype errors, nor which model best fits the real WGS data generated from the missing persons samples. Thus, the commonly adopted simulation tool, Ped-Sim was used, which a pragmatic solution with one single parameter and the simulations could be better controlled on a reasonable scale*.* Nevertheless, the classifier trained by the simulated data still outperformed the individual measures. The more sophisticated simulation methods will be tested in future studies.

It is relatively easy to differentiate parent-child and full-sibling within the 1st degree. The combination of IBS0 and K1 could reach 100% accuracy when GERs were less than 0.05. But it dropped quickly when the GER was higher than or equal to 0.05 (Figure 6A). With the selected features, the classification accuracy could reach 100% across all the genotyping error levels. However, the accuracies for estimating the relationship within the 2nd and 3rd degrees were much lower, which was consistent with previous studies (Epstein et al., 2000; Huff et al., 2011; Henn et al., 2012; Ramstetter et al., 2017). In particular, the accuracies of estimating relationships could be equivalent to random guessing (i.e., 33 and 25% within the 2nd and 3rd degree, respectively), if the genotyping errors in the training dataset and the test set were largely different (e.g., 0 for the training dataset and 0.1 for the test set). With more accurate genotyping error estimations, the classifier can be trained with more proper data (i.e., the data with the same GERs as the test set), and the classification accuracy could be improved.

The genotyping errors could come from every step of the genotyping or sequencing process, including the polymerase chain reaction (PCR), sequencing chemistry, hybridization, signal detection, data collection, base-calling, sequence alignment, variant calling, etc. The GER would depend on the quantity and quality of the samples, the genotyping or sequencing protocols, and the bioinformatics analysis pipeline(s). Data cleaning in the bioinformatics analysis (e.g., removing sequence reads with low-quality scores) could reduce the GER with the cost of losing variants. Fortunately, our study and Shcherbina’s study (Shcherbina et al., 2016a; Shcherbina et al., 2016a) showed that losing 90% of the SNPs in the GSA panel did not affect the estimation accuracy. Thus, a stringent data cleaning process could be implemented to remove low-quality data and lower the GERs. In addition, a method to precisely estimate the GER of a SNP profile, or at least a range of the GERs with confidence levels, would have practical value (but is yet to be developed).

In this study, two strong classifiers, RF and SVM, were tested. However, more recently developed classification algorithms, such as XGBoost (Chen & Guestrin, 2016) and deep learning (LeCun et al., 2015), may further improve the performance with proper parameter tuning. It is also worth noting that feature selection is very important to increase classification accuracy. The 17 features listed in Supplementary Table S1 were collected from previous literature, and some of these features might be noisy for certain relationships or relationship degrees. Using the feature selection in relationship degree as an example, when the features were added one by one, the accuracy experienced three stages, raising, platform, and falling. It showed that some features increased the classification accuracy, but some features may be irrelevant, noisy, and even reduce the accuracies for certain relationship degrees or types, partially due to the genotyping errors.

This current method does not require allele frequencies as input, which is the case for many missing person cases. Additional features based on allele frequencies (e.g., the cumulative likelihood of observing a SNP profile given a specific population) may be included in a future study, as allele frequencies of the SNPs could provide more information than just SNP genotypes, and thus higher accuracy could be achieved by combining the features based on genotypes and features based on allele frequencies. However, the effect of inaccurate allele frequencies (e.g., use of African frequencies for Hispanic samples) is yet to be investigated. The features on IBD segments (e.g., the average length of the IBD segments, the total length of the IBD segments, etc.) (Hill & White, 2013; Ramstetter et al., 2018) may not work well with high genotyping errors, as the segments could be easily interrupted by the errors. However, those IBD segment features could be included for cases with negligible errors (i.e., cases with high quality and quantity samples). The GEDmatch results of real data showed that the total length of IBD segments could accurately identify 1st, 2nd, and 3rd pedigree degrees using the samples with low-level genotyping errors (i.e., samples with 50 ng DNA). However, the majority of the related pairs were not detected with GEDMatch when the genotyping error rates were high (i.e., samples with 500 and 100 pg DNA). In addition, in many missing persons cases, the samples’ ancestry information may not be available or precisely determined. If the sample is admixed or belongs to an admixture population, the genome-wide relatedness methods such as KING will lead to bias estimation (Thornton et al., 2012; Conomos et al., 2016). The performance of this machine learning approach has not been tested in the admixed population as only European samples were involved in our study. The effect of the admixture population will be considered in our future method development.

To summarize, a novel machine learning-based approach was developed in this study to combine multiple measures and estimate the relationships for profiles with high GERs. Substantial accuracy increase and robustness improvement were observed in determining both relationship degrees and relationship types, which imply that the machine learning approach can increase the robustness of relationship estimations. Further improvement may be conducted by combining more features based on allele frequencies and IBD segments.

## Data Availability

The original contributions presented in the study are publicly available. This data can be found here: GSE209804.
