# Supplementary material for: A machine learning approach for missing persons cases with high genotyping errors
Source: Front Genet. 2022 Oct 3;13:971242. doi: 10.3389/fgene.2022.971242 (PMC9573995; doi:10.3389/fgene.2022.971242)
Supplement: Supplementary file 2 [file Table1.DOCX]

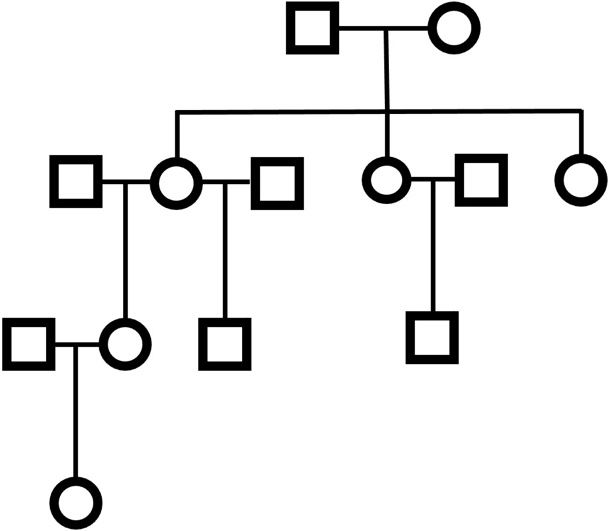


Figure S1. The designed pedigree for simulation. The number of individual pairs of each relationship type in this pedigree was listed in Table 1.

Table S1. The list of 17 features of relatedness.

| Name | Description |
| --- | --- |
| K1 (Manichaikul et al., 2010) | KING-robust measure in KING software |
| K0 (Manichaikul et al., 2010) | KING-homo measure in KING software |
| IBS0 (Stevens et al., 2011) | The proportions of sharing 0 IBS allele |
| IBS1(Stevens et al., 2011) | The proportions of sharing 1 IBS allele |
| IBS2 (Stevens et al., 2011) | The proportions of sharing 2 IBS alleles |
| IBS01 (Stevens et al., 2011) | IBS0 + IBS1 |
| IBS02 (Stevens et al., 2011) | IBS0 + IBS2 |
| IBS12 (Stevens et al., 2011) | IBS1 + IBS2 |
| j1 (Waples et al., 2019) | The proportion of sharing 2 reference alleles in both individuals A and B |
| j2 (Waples et al., 2019) | The proportion of sharing 1 reference allele in individual A and sharing 2 reference alleles in individual B |
| j3 (Waples et al., 2019) | The proportion of sharing 0 reference allele in individual A and sharing 2 reference alleles in individual B |
| j4 (Waples et al., 2019) | The proportion of sharing 2 reference alleles in individual A and sharing 1 reference alleles in individual B |
| j5 (Waples et al., 2019) | The proportion of sharing 1 reference allele in both individuals A and B |
| j6 (Waples et al., 2019) | The proportion of sharing 0 reference allele in individual A and sharing 1 reference allele in individual B |
| j7 (Waples et al., 2019) | The proportion of sharing 2 reference alleles in individual A and sharing 0 reference allele in individual B |
| j8 (Waples et al., 2019) | The proportion of sharing 1 reference allele in individual A and sharing 0 reference allele in individual B |
| j9 (Waples et al., 2019) | The proportion of sharing 0 reference allele in both individuals A and B |
