# Supplementary figures and images for: A machine learning approach for missing persons cases with high genotyping errors
Source: Front Genet. 2022 Oct 3;13:971242. doi: 10.3389/fgene.2022.971242 (PMC9573995; doi:10.3389/fgene.2022.971242)

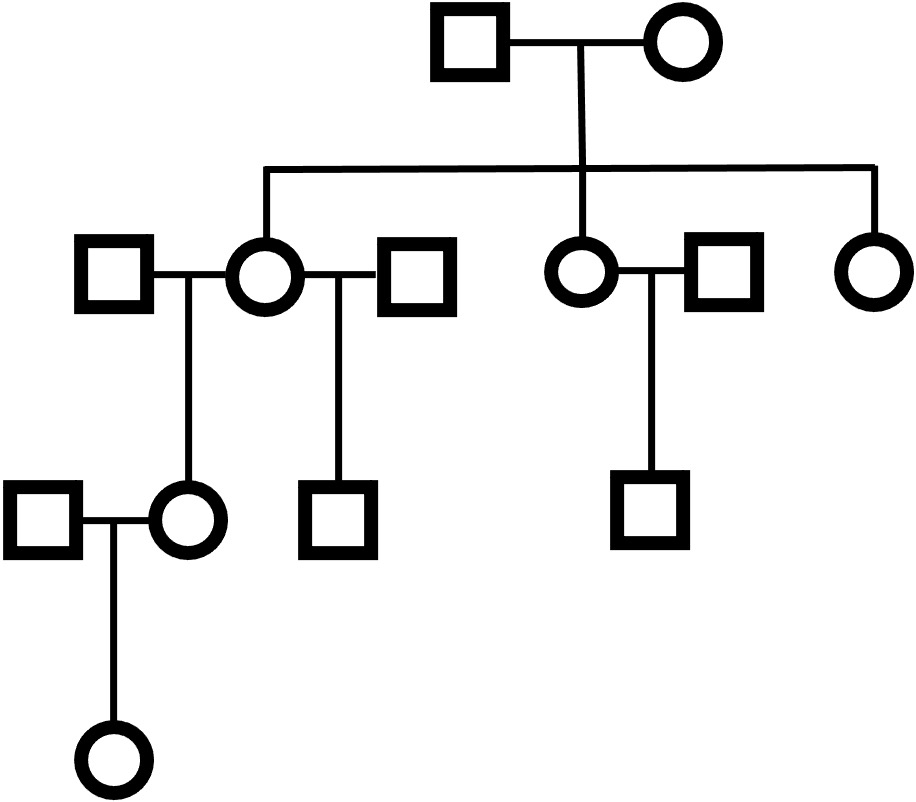

Supplement: Supplementary file 3 [file Image1.JPEG]
